# Life After Stroke and Supportive Stroke Pathways: Protocol For A Rapid Realist Review

**DOI:** 10.12688/hrbopenres.14034.1

**Published:** 2025-01-23

**Authors:** Olive Lennon, Mary O'Neill, Killian Walsh, Frances Horgan

**Affiliations:** 1 University College Dublin, School of Public Health Physiotherapy, and Sports Science, Dublin, Leinster, Ireland; 2 School of Physiotherapy, Royal College of Surgeons in Ireland, Dublin, Leinster, Ireland; 3 Royal College of Surgeons in Ireland, Mercer Library, Dublin, Leinster, Ireland

**Keywords:** Life after stroke; Realist review; Programme theory; Supportive pathways

## Abstract

**Background:**

Ever-growing numbers of individuals are surviving stroke and living with the consequences. Life after stroke is a key pillar in addressing the burden of stroke for the remaining lifespan for those with stroke. No consensus on how to best promote agency and fulfilment in life after stroke or the resources required to achieve this currently exists.

**Methods:**

In this realist review protocol we outline the methods we will use to gain an understanding of supporting Life after Stroke through the development of programme theories. These will consist of context–mechanism–outcome configurations (CMOCs) and will acknowledge the resources required. The review will follow the RAMESES five-stage structured methodology to (1) define the scope of the review, and the development of initial programme theories for supporting life after stroke, (2) develop a comprehensive search strategy to identify relevant research, (3) review primary studies and extract data, (4) synthesise evidence (5) refine programme theories iteratively throughout the process using an Expert Panel and reference group, to including stroke researchers, health care professionals working in stroke care and stroke support agencies.

**Conclusion:**

This realist review aims to conceptualise supports for Life after Stroke. The CMOCs developed will help explain how generative causation within the life after stroke pathway works. The findings will help inform policy and practice and inform future realist evaluations of Life after Stroke support pathways.

## Introduction

Stroke is a leading cause of death and disability, affecting survivors physically, emotionally, cognitively and socially, and impacting their wider family and social networks ^1– 5^. In 2022 in Ireland, 5961 adults were admitted to acute hospitals following stroke, a year-on-year increase since 2019 ^6^. Sixty-six per cent of individuals discharged home, had documented mild to moderate stroke-related disability. By 2035 there will be a 34% increase in stroke events in the EU, and by 2047, the projected rise in prevalent cases in the community is 27% ^7, 8^.

There is no consensus on what is meant by best care following discharge from specialist stroke services ^1^. The Burden of Stroke in Europe Report describes life after stroke under four categories: health and activity issues, adjustment and wellbeing and information and support ^7, 8^. Multiple unmet needs after stroke have been described in the literature including body function and structure, activities, participation, environmental and personal domains ^8– 10^. Published Irish data on unmet needs identified mobility problems, emotional issues, fatigue, concentration difficulties, negative financial changes and low return-to-work rates after stroke ^11^.

A qualitative synthesis described the disruption to life experienced by people and their families and the need to adapt and rebuild a post-stroke life and identity ^12^. Documented post-stroke experiences included autonomy, uncertainty, engagement, hope and social relations as key challenges for participation in life after stroke ^8, 9, 13^.

The Stroke Action Plan for Europe (SAPE) (2018–2030) recognises Life after Stroke as one of seven key pillars to address the burden of stroke and long-term consequences ^1^. The SAPE general principles for Life after Stroke and the World Stroke Organisation include a call for the recognition by society, of the worth and value of people with stroke-related disabilities ^14– 17^.

Our rapid realist review seeks to identify the mechanisms that promote agency and fulfilment in life after stroke and the resources required to achieve this. We will explore the contexts in which interventions have been successful, and will include the views and opinion of stakeholders in building programme level theories. This realist review will ask “what works for whom, why does it work and in what circumstances?” ^18^.

## Protocol

### Study design

Realist synthesis is a theory driven approach and realist philosophy is based on ontological assumptions of a “real world” ^19^. A realist lens conceptualises services and interventions that support Life after Stroke as a dynamic process ^19, 20^. Context-mechanism-outcome configurations (CMOCs) explain generative causation, what mechanisms are activated and how these interactions lead to different outcomes ^21, 22^. Our review will be conducted and reported according to the Realist and Meta-narrative Evidence Syntheses: Evolving Standards (RAMESES) ^22^. The PRISMA-P checklist will be used ^23^.

## Methods

### Stages of the realist review

**Figure 1.**
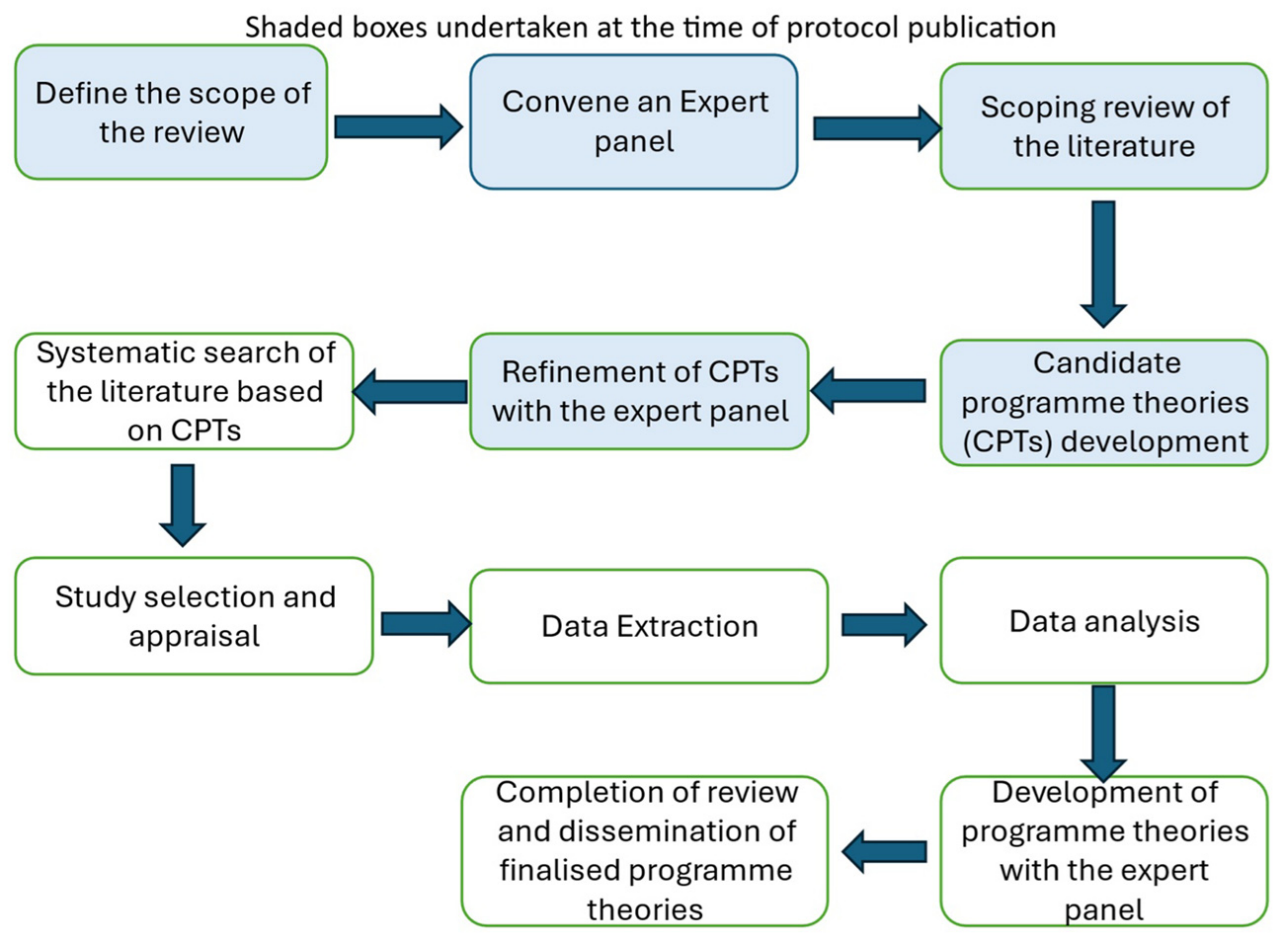
Five stages of the realist review in accordance with Pawson’s work ^24^.

Figure 1 outlines the five stages of the realist review in accordance with Pawson’s work ^24^.

### Defining the scope of the review

The aim of this realist review is to describe the mediators which enable a personally meaningful life after stroke. By determining the contexts, mechanisms and resources, the results will help guide clinicians, healthcare providers and peers to support people after stroke to regain a sense of identity, purpose, fulfilment and life satisfaction. The specific research questions being asked include:

*What mediators or mechanisms enable interventions designed to support a personally meaningful life after stroke to result in the anticipated outcomes?*

*What contextual factors and resources help facilitate achievement of a personally meaningful life after stroke following supportive interventions?*

### The expert panel

An expert panel was convened including clinicians, researchers, a recognised international expert in the field, healthcare professionals working in stroke care, advocacy, stroke support workers from NGOs and people with lived experience of stroke. Table 1 provides a breakdown of the members of the expert group. This group will have an important role, placing a spotlight on key literature, providing lived experience as someone who works in stroke care or has personal experience of stroke to aid the interpretation of data and complex concepts, in assessing the available evidence and in enhancing the credibility of the programme-level theories developed and their relevance clinically.

**Table 1.** The Expert Panel membership.

Membership N=13	Gender breakdown	Sector	Additional information
National Stroke Programme representation (N=2)	Female: 2	Health Service Executive	Responsibilities of the programme include development of the National Stroke Strategy
People with lived experience of stroke (N=2)	Female: 1 Male: 1	Not applicable	
National stroke charity representation (N=2)	Female: 1 Male: 1	NGO	Advocacy director & Head of community support services
Physiotherapy (N=2)	Female: 2	Academic Clinical	
Occupational therapy (N=2)	Female: 1 Male: 1	Academic Clinical	
Speech and language therapy (N=1)	Female: 1	Clinical	
Psychology (N=1)	Female: 1	Clinical	
Nursing (N=1)	Female: 1	Academic	International Expert

### Early programme theory development

Potential explanations for how supports to achieve a personally meaningful Life after Stroke might work are first required. Describing candidate programme theories (CPTs) for a realist review is an important step, and will provide a scaffold on which the causal mechanisms can generate outcomes in specific contexts ^25^. This step will begin with scoping the existing literature and describing early hypotheses about an intervention’s mechanism of action and expert-level insights ^26, 27^. A preliminary search of CINAHL and PubMed by title, abstract, keywords and subject headings was conducted in conjunction with a liaison librarian using search strings derived from the PICOC headings detailed in Table 2.

**Table 2.** PICOC Defined and Inclusion and Exclusion criteria.

	Inclusion Criteria	Exclusion Criteria
**P**	Adult stroke or cerebrovascular accident or transient ischaemic attack.	Other neurological conditions including traumatic brain injury. Paediatric stroke.
**I**	Interventions designed to practically or psychologically support someone with stroke to have a personally meaningful life after stroke. Interventions may include self-management, peer support, communication, living well and healthily, goal setting and resilience building.	Studies focussed on acute medical interventions, pharmacotherapy, rehabilitation of stroke impairments and disability.
**C**	Not required but studies could include usual care or same or other interventions as comparator groups.	
**O**	Adapting to loss after stroke; re-establishing a self-identify and self-confidence; engaging in work (vocational or other), engaging in family and societal roles; managing psychological stress; purpose and fulfilment after stroke; wellbeing.	Impairment and disability outcomes.
**C**	Following discharge from acute hospital services across the remaining lifespan.	Studies in acute hospital or in-patient rehabilitation settings. Studies not in the English language.

This guided the first group of CPTs developed. Consultation between the researchers provided a first level refinement of these CPTs, often collapsing CPTs or separating them out where differing constructs were determined. These were then presented to the expert panel and further refined, taking account of their perspectives and experiences in relation to contexts, mechanisms and outcomes of supports for Life after Stroke. Here the panel were tasked with confirming, refuting or refining the CPTs ^23^.

### Development of a search strategy

The broad search terms derived from the CPTs are summarised in Table 3 and will be developed further with a liaison librarian, aligned to the RAMESES guidance ^22, 23, 28^.

**Table 3.** Candidate Programme Theories.

Candidate programme theory	Theme	Early COMCs
**1**	**Supported self-management after stroke**	Having agency and access to supportive resources when and as required from healthcare professionals and or family and or peers to manage life after stroke creates a space and a support system in which stroke survivors can feel comfortable to flourish and work towards their hopes and priorities to achieve a personally meaningful life after stroke.
**2**	**Goals and priorities and identifying needs after stroke**	When transitioning from active rehabilitation the conversation should move from shared rehabilitation goals to a more skilled conversation, in collaboration with services and therapists, that establishes their readiness for life after stroke, what is a priority for the person with stroke and their family, what is important to them and what they need to re-establish a sense of identity, promote adjustment to the new normal and allow active citizenship that may include engaging in work, vocational or other endeavours.
**3**	**Peer support**	For those who wish to engage with it, formal peer support with resources and training; or adequately resourced informal peer support or incidental peer support can all play an important role in adjustment to life after stroke by providing a safe space to talk, promoting self-compassion and perspectives of gratitude, decreasing isolation and increasing a sense of connectedness and fulfilment in the community.
**4**	**Communication**	Ongoing communication that includes active listening and a skilled communication partner (or specific supports for those with language difficulty after stroke e.g. speech and language therapist facilitated), allows the person with stroke and their wider support network (or nominated supporter) to air their feelings and express complex emotions or trauma, to communicate their hopes for their future life, to seek the help they require and to re-engage in activities after their stroke that are meaningful for them.
**5**	**Psychological supports**	Psychological supports, that can range from and include; professional support, to talk therapy, to active listening; can provide ‘headspace’ that helps people with stroke to work through complex emotions and internal thoughts, to develop a compassionate view of themselves, helps them to restore their locus of control and ability to seek help, leading to improved perceptions of well-being and self-esteem in their life after stroke.
**6**	**Living well after stroke**	In individuals who have an understanding of risk factors for stroke (these can include medication adherence, physical activity, participation, healthy dietary habits, smoking cessation, safe substance use, alcohol and other emotional self-regulation and mood), self-evaluation of risk and self-directed choices should be supported by positive messaging and nudges for those who wish to change unhealthy lifestyle behaviours. Adequately resourced services, with supports for behaviour change, underpinned by behaviour change theory and techniques including monitoring and feedback, can positively impact cardiovascular health and wellness.

Multiple sources including electronic databases, organisational websites and grey literature will be searched and we will cross referencing citations in identified articles and suggested sources from the expert panel. The following databases CINAHL, EBSCOhost, MEDLINE, PubMed, EMBASE, Web of Science, Scopus, ScienceDirect Journals, and Google Scholar will be searched to identify the studies relevant to the review questions. The searches will include combinations of the keywords based on the population-intervention-comparison-outcome-context (PICOC) tool. This tool will help to describe the inclusion and exclusion criteria. An indicative search strategy for Ovid Medline is included ( Table 4).

**Table 4.** Indicative Search Strategy.

*No.*	*Query*	*Run Via*	*Results*
1	(TI=(stroke or cardiovas* or "cerebral infarc*" or cerebral or "acquired brain injur*" or infract* or intracereb* or "cerebrovascular accident*" or "CVA" or "cerebrovascular insult" or "CVI" or "brain attack")) OR AB=(stroke or cardiovas* or "cerebral infarc*" or cerebral or "acquired brain injur*" or infract* or intracereb* or "cerebrovascular accident*" or "CVA" or "cerebrovascular insult" or "CVI" or "brain attack")	Web of Science	1,269,283
2	(TI=("integrated care" or "ICS" or "mulit-disciplinary care" or "multidisciplinary care" or "coordinated care" or "co-ordinated care" or "fragmentation of care" or "continuity of care" or "transition of care" or "integrated health" or "comprehensive care" or "seamless care" or "transmural care" or "collaborative care" or "team based care" or "team-based-care" or "MDT" or ((team or multidisciplinary or "multi disciplinary") NEAR/2 care) )) OR AB=("integrated care" or "ICS" or "mulit-disciplinary care" or "multidisciplinary care" or "coordinated care" or "co-ordinated care" or "fragmentation of care" or "continuity of care" or "transition of care" or "integrated health" or "comprehensive care" or "seamless care" or "transmural care" or "collaborative care" or "team based care" or "team-based-care" or "MDT" or ((team or multidisciplinary or "multi disciplinary") NEAR/2 care ))	Web of Science	89,274
3	(TI=("care planning" or "social support*" or "need* assessment*" or "access to care" or "service sign* post*" or "recreational activit*" or "recreational hobb*" or "social support" or "self management" or "recreational hobb*")) OR AB=("care planning" or "social support*" or "need* assessment*" or "access to care" or "service sign* post*" or "recreational activit*" or "recreational hobb*" or "social support" or "self management" or "recreational hobb*")	Web of Science	133,521
4	(TI=((peer NEAR/2 social) or (care NEAR/2 transition) or (pathway* NEAR/2 care) or (support* NEAR/2 group*) or (service NEAR/2 sign*) or ((physical or psychological) NEAR/2 support*)) OR AB=((peer NEAR/2 social) or (care NEAR/2 transition) or (pathway* NEAR/2 care) or (support* NEAR/2 group*) or (service NEAR/2 sign*) or ((physical or psychological) NEAR/2 support*)))	Web of Science	90,290
5	(TI=("universal access*" or 'primary screening' or ((multisectoral or multi-sectoral) NEAR/2 "public health intervention*") or "patient focused care" or "chain of care" or ((individual or societ*) NEAR/2 prevention*) or "evaluation of outcome*" or "primary prevention initiative*" or (target NEAR/2 "whole population*") or "self management*" or "health* lifetime change*" or "personalized assessment*" or "universal access" or "national strateg*" or "public health campaign*" or ((legislation* or national) NEAR/2 strateg*) or "early supported discharge" or (early NEAR/2 "supported discharge") or "specialist knowledge" or (skilled N2 "stroke personnel") or "evidence based pathway*" or "national stroke plan*" or "coordinated care" or "co ordinated care" or "co-ordinated care" or ((optimised or optimized or equal) N3 "access care") or (onset N2 treatment*) or "thrombolysis" or "specialist advice" or "continuum of care" or "specialist rehabilitation" or "early supported discharge" or (ongoing NEAR/3 intensive NEAR/2 intervention*) or "lifestyle support*" or "physical fitness program*" or (review NEAR/2 need*) or (adopting NEAR/2 "local condition*") or "patient centered care" or "patient centred care" or (national NEAR/3 "stroke care") or "enhanced self management*" or "enhanced self-management" or "assistance system*" or "secondary prevention" or (secondary NEAR/2 prevention) )) OR AB=("universal access*" or 'primary screening' or ((multisectoral or multi-sectoral) NEAR/2 "public health intervention*") or "patient focused care" or "chain of care" or ((individual or societ*) NEAR/2 prevention*) or "evaluation of outcome*" or "primary prevention initiative*" or (target NEAR/2 "whole population*") or "self management*" or "health* lifetime change*" or "personalized assessment*" or "universal access" or "national strateg*" or "public health campaign*" or ((legislation* or national) NEAR/2 strateg*) or "early supported discharge" or (early NEAR/2 "supported discharge") or "specialist knowledge" or (skilled N2 "stroke personnel") or "evidence based pathway*" or "national stroke plan*" or "coordinated care" or "co ordinated care" or "co-ordinated care" or ((optimised or optimized or equal) N3 "access care") or (onset N2 treatment*) or "thrombolysis" or "specialist advice" or "continuum of care" or "specialist rehabilitation" or "early supported discharge" or (ongoing NEAR/3 intensive NEAR/2 intervention*) or "lifestyle support*" or "physical fitness program*" or (review NEAR/2 need*) or (adopting NEAR/2 "local condition*") or "patient centered care" or "patient centred care" or (national NEAR/3 "stroke care") or "enhanced self management*" or "enhanced self-management" or "assistance system*" or "secondary prevention" or (secondary NEAR/2 prevention) )	Web of Science	235,698
6	TI=(therap* or ("mental health" NEAR/2 (support* or therap* or service*)) or counselling or rehabilitation or (wellbeing NEAR/2 support) or "behavioral therapy" or "behavioural therapy" or "residential care" or "crisis intervention*" or "individual care plan*" or "social worker*" or "mental health treatment*" or "wellbeing support*" or "well-being support*" or "psychiatric support*" or "emotional support*" or ("self-efficacy" NEAR/2 support) or therap* or (("self-efficacy" or autonomy) N2 support))	Web of Science	1,279,103
7	AB=(therap* or ("mental health" NEAR/2 (support* or therap* or service*)) or counselling or rehabilitation or (wellbeing NEAR/2 support) or "behavioral therapy" or "behavioural therapy" or "residential care" or "crisis intervention*" or "individual care plan*" or "social worker*" or "mental health treatment*" or "wellbeing support*" or "well-being support*" or "psychiatric support*" or "emotional support*" or ("self-efficacy" NEAR/2 support) or therap* or (("self-efficacy" or autonomy) N2 support))	Web of Science	3,004,968
8	#2 OR #3 OR #4 OR #5 OR #6 OR #7	Web of Science	4,125,431
9	(TI=(recovery or cognit* or neuropsych* or "mild cognitive impairment*" or "vascular mild cognitive impairment*" or attention or attention* or "information process*" or memory or recall or executive or dysexecutive or memory or "executive function*" or "attention*")) OR AB=(recovery or cognit* or neuropsych* or "mild cognitive impairment*" or "vascular mild cognitive impairment*" or attention or attention* or "information process*" or memory or recall or executive or dysexecutive)	Web of Science	3,719,350
10	TI=(negativity or (negative NEAR/2 (feeling* or emotion* or thought* or "mental state*")) or depression or depressive or melancholy or unhappiness or sorrow or woe or "poor mental state*" or "wellbeing" or "well-being" or melancholy or sadness or unhappiness )	Web of Science	308,614
11	AB=(negativity or (negative NEAR/2 (feeling* or emotion* or thought* or "mental state*")) or depression or depressive or melancholy or unhappiness or sorrow or woe or "poor mental state*" or "wellbeing" or "well-being" or melancholy or sadness or unhappiness )	Web of Science	700,256
12	TI=(work* or occupat* or "paid employment*" or employ* or vocation* or parent* or caregiv* or carer* or caring or mother* or father* or relationship* or relation or relations or caregiv* or leisure* or sport* or ("life participation" NEAR/3 (participation or engagement* or role)))	Web of Science	2,849,953
13	AB=(work* or occupat* or "paid employment*" or employ* or vocation* or parent* or caregiv* or carer* or caring or mother* or father* or relationship* or relation or relations or caregiv* or leisure* or sport* or ("life participation" NEAR/3 (participation or engagement* or role)))	Web of Science	11,930,236
14	#9 OR #10 OR #11 OR #12 OR #13	Web of Science	16,421,368
15	((TI=("long term" or "longterm" or "long-term")) OR AB=("long term" or "longterm" or "long-term"))	Web of Science	1,436,868
16	#1 AND #8 AND 14 AND 15	Web of Science	9,576
17	(TI=("middle age" OR "young adult" OR "working age" OR "working adult*")) OR AB=("middle age" OR "young adult" OR "working age" OR "working adult*")	Web of Science	57,013
18	#16 AND #17	Web of Science	***61***
19	(TI=("middle age" OR "young adult" OR "working age" OR "working adult*")) OR AB=("middle age" OR "young adult" OR "working age" OR "working adult*")	Web of Science	59,547
20	#16 AND #20	Web of Science	***70***
21	#20 AND #16 and 2013 or 2014 or 2015 or 2016 or 2023 or 2022 or 2021 or 2020 or 2019 or 2017 or 2018 (Publication Years) and English (Languages)	Web of Science	***54***

### Evidence selection and appraisal

Papers will be included based on their relevance to the research question and IPTs ^19^. We will assess the relevance and rigor of included works as we test the IPTs ^29^.

### Data extraction

Data extraction will be a three-step process, initial title and abstract screening using the inclusion and exclusion criteria ( Table 2); followed by full-text review and appraisal. All authors will screen documents for initial relevance. Any conflicts will be discussed by all authors. A bespoke data extraction form will be used to extract and review information for programme theory refinement to include context, mechanisms, outcomes and resources relating to the research questions.

### Data analysis

Following data extraction, the selected papers will be imported into the qualitative software NVivo [91] version 14 (Mac), to facilitate coding and thematic data analysis to identify CMOCs. The expert panel will help to improve the final theoretical framework and we will revisit any stage of the process to ensure that we have enough data and reach a state of ‘theory’.

### Refinement of Initial Programme Theories (IPTs)

The first draft IPTs will be presented to the expert panel for feedback to ensure appropriate interpretation of results. The expert panel will ensure that the refined IPTs are robust, contextually appropriate and reflect the complexities associated with the implementation of the intervention and relevant outcomes ^25^.

### Dissemination of findings

Dissemination will be consistent with the RAMESES guidelines ^22, 28^. The findings will be published in a peer-reviewed journal, presented at relevant conferences and stakeholder events. The protocol will be registered with PROSPERO.

## Discussion

Realist inquiry facilitates an understanding of the extent to which an intervention works, for whom, in what context, why and how. This methodology has previously proven useful in research relating to stroke care in the context of early supported discharge ^30^, neuropsychology rehabilitation ^31^ and in evaluating outpatient rehabilitation and community support services post stroke ^32^. This protocol adds to the growing number of publications in stroke care. It provides details for a realist review that will enable a better understanding of the specific contexts in which certain mechanisms are activated to enable long-term support to achieve a personally meaningful life after stroke, targeting both the visible and often hidden forces that generate the outcomes of interest ^32, 33^.

Life after stroke has only been regarded as a separate entity in recent years. Few of the current adult UK guidelines specifically address longer term stroke management ^34^, reflecting the paucity of evidence in this area and the limited programme theory available to support the required complex interventions. The realist review proposed in this protocol, rather than judging the effectiveness of an intervention, is concerned with answering how supports provided for Life after Stroke work, who they work for and in what circumstances.

Realist programme theories that can coherently explains how supports are expected to work in Life after Stroke will provide researchers, policy makers and service funders/providers with new insights and robustly generated programme theories that can be developed, implementation or evaluated in existing stroke care pathways. Key to the knowledge gain associated with this methodologic approach is the inclusion of reference panels comprising healthcare professional and experience-based experts in the development of the research questions, the search strategy and in the refinement of the review’s findings to both an Irish and a personal context.

## Ethics and consent

Ethical approval and consent were not required.

## Data Availability

No data are associated with this protocol. Open Science Framework: CLASP project - Life after stroke and supportive stroke pathways: Protocol for a rapid realist review. https://doi.org/10.17605/OSF.IO/UJC5X ^35^. The project contains the following underlying data: Extended File 1: PRISMA-P – complete checklist of items in reporting scoping reviews Extended File 2: Search Terms – complete list of search terms used in all databases Data are available under the terms of the Creative Commons Attribution 4.0 International license (CC-BY 4.0).
