# Supplementary material for: What are the Mechanisms and Contexts for a Supported Life After Stroke Pathway: Protocol for a Rapid Realist Review
Source: HRB Open Res. 2025 Jul 14;8:10. Originally published 2025 Jan 23. [Version 3] doi: 10.12688/hrbopenres.14034.3 (PMC11986413; doi:10.12688/hrbopenres.14034.3)
Supplement: Peer review history

# Reviewer report: Reviewer response for version 3

**DOI:** 10.21956/hrbopenres.15617.r48350

**Published:** 2025-08-27

**Authors:** Rita Chiaramonte

**Affiliations:** 1 University of Catania, Catania, Italy

Thank you for the opportunity to review this protocol for a rapid realist review aimed at conceptualizing support for life after stroke—an under-researched area in the stroke care pathway.

 I appreciated the efforts of the authors to improve their study according to the suggestions of the reviewers.

 The protocol closely follows RAMESES methodological guidance and appropriately uses context–mechanism–outcome configurations to develop programme theories aligned with the study’s objectives.

Is the study design appropriate for the research question?

Yes

Is the rationale for, and objectives of, the study clearly described?

Partly

Are sufficient details of the methods provided to allow replication by others?

Yes

Are the datasets clearly presented in a useable and accessible format?

Yes

Reviewer Expertise:

Rehabilitation

I confirm that I have read this submission and believe that I have an appropriate level of expertise to confirm that it is of an acceptable scientific standard.

# Reviewer report: Reviewer response for version 2

**DOI:** 10.21956/hrbopenres.15524.r46546

**Published:** 2025-04-10

**Authors:** Diane Trusson

**Affiliations:** 1 University of Nottingham, Nottingham, UK

Thank you for the opportunity to review this manuscript. It is good to see this very important, yet under-researched,  topic within stroke research being addressed. It seems that the manuscript is much stronger as a result of the revisions made in response to the previous reviewer's comments. Consequently I have no further recommendations in terms of the methodology for the proposed realist review. 

 The only comment I have is in terms of readability. There are a number of long, complex sentences which would be clearer if they were broken up. Specifically the following:

 Page 3 - Second sentence under the heading 'the expert panel'.

 Page 6 -The entries under the heading 'Early COMCs' in table 3.

 Page 8 - Last sentence in the first paragraph of the discussion.

 I would also recommend that the authors proofread the manuscript before it is submitted.

 Overall I congratulate the authors on this work and look forward to reading the realist review in due course.

Is the study design appropriate for the research question?

Yes

Is the rationale for, and objectives of, the study clearly described?

Yes

Are sufficient details of the methods provided to allow replication by others?

Yes

Are the datasets clearly presented in a useable and accessible format?

Yes

Reviewer Expertise:

NA

I confirm that I have read this submission and believe that I have an appropriate level of expertise to confirm that it is of an acceptable scientific standard.

# Reviewer report: Reviewer response for version 2

**DOI:** 10.21956/hrbopenres.15524.r46543

**Published:** 2025-04-07

**Authors:** Rita Chiaramonte

**Affiliations:** 1 University of Catania, Catania, Italy

The study aims to identify consensus on best practices for care following discharge from specialist stroke services.

**Title:**

 The current title is unclear. Please revise it to more explicitly reflect the aim and methodology of the study. The title should clearly inform the reader about the scope and purpose of the manuscript.

**Abstract:**

 The aim of the study is currently unclear in the abstract. This will help readers quickly understand the intent and contribution of the work.

**Table 3: **Table 3 would benefit from more detailed discussion and contextualization in the main text. This will enhance the reader’s understanding and make the manuscript more engaging. Please provide more narrative explanation in the text that connects to the contents of Table 3.

 Consider incorporating references to support the relevance of each theme listed in the table. For example, for the theme “Communication”, see the following: DOI: 10.23736/S1973-9087.20.06242-5

 Table 3 represents the developmental framework for your next study. This should be emphasized more clearly, highlighting that while the literature discusses these themes, it rarely does so in the context of post-discharge care—and not as comprehensively as your study aims to do.

Is the study design appropriate for the research question?

Yes

Is the rationale for, and objectives of, the study clearly described?

Partly

Are sufficient details of the methods provided to allow replication by others?

Yes

Are the datasets clearly presented in a useable and accessible format?

Yes

Reviewer Expertise:

Rehabilitation

I confirm that I have read this submission and believe that I have an appropriate level of expertise to confirm that it is of an acceptable scientific standard, however I have significant reservations, as outlined above.

## References

[1] : Dysarthria and stroke. The effectiveness of speech rehabilitation. A systematic review and meta-analysis of the studies. *Eur J Phys Rehabil Med* .2021;57(1) : 10.23736/S1973-9087.20.06242-5 24-43 10.23736/S1973-9087.20.06242-5 32519528

# Reviewer report: Reviewer response for version 2

**DOI:** 10.21956/hrbopenres.15524.r46508

**Published:** 2025-03-27

**Authors:** Nicola Hancock

**Affiliations:** 1 University of East Anglia, Norwich, UK

No new comments

Is the study design appropriate for the research question?

Yes

Is the rationale for, and objectives of, the study clearly described?

Yes

Are sufficient details of the methods provided to allow replication by others?

Partly

Are the datasets clearly presented in a useable and accessible format?

Not applicable

Reviewer Expertise:

Stroke rehabilitation, technology for rehabilitation, stroke service innovation

I confirm that I have read this submission and believe that I have an appropriate level of expertise to confirm that it is of an acceptable scientific standard.

# Reviewer report: Reviewer response for version 1

**DOI:** 10.21956/hrbopenres.15406.r45178

**Published:** 2025-03-17

**Authors:** Nicola Hancock

**Affiliations:** 1 University of East Anglia, Norwich, UK

Thank you for the opportunity to review this protocol for a rapid realist review that aims to conceptualise support for Life After Stroke- this is an under-researched part of the stroke pathway and, according to my interpretation of the protocol,  the review will make a valuable contribution to understanding what works for whom and in what context, after discharge from statutory stroke services. 

 It is pleasing to see that the protocol closely adheres to RAMESES methodological guidance and seeks to develop programme theories using context-mechanism-outcome configurations, which are appropriate to the intended aims of the study. As such, i have only minor suggestions for improvement, which i hope are useful to the authorship team:

 - It is important that people with lived experience are included in the summary of expert panel members in the abstract. i appreciate that their involvement is clearer later on in the manuscript (table 1), but a commitment to the full range of stakeholders should be explicit at the outset.

 -The review question, in the second paragraph of page 3, is  general and does not include any reference to the population or context of life after stroke. Perhaps the addition of 'for people in their life after stroke' or similar would help here? 

 - A minor point, but suggest figure 1 legend simply says 'According to Pawson (ref) rather than "Pawson's work' 

 -the commitment to stakeholder engagement is laudable in the protocol, but i see no reference to carers. Carers are key stakeholders in life after stroke but seem to be absent from the expert panel- perhaps they will be included/represented by the SSOs but this could be clearer. 

 -the search strategies are very comprehensive

 -a brief expansion of detail of the approach to coding and thematic analysis in the data analysis would enhance transparency and the interpretation of future work reporting the full study.

Is the study design appropriate for the research question?

Yes

Is the rationale for, and objectives of, the study clearly described?

Yes

Are sufficient details of the methods provided to allow replication by others?

Partly

Are the datasets clearly presented in a useable and accessible format?

Not applicable

Reviewer Expertise:

Stroke rehabilitation, technology for rehabilitation, stroke service innovation

I confirm that I have read this submission and believe that I have an appropriate level of expertise to confirm that it is of an acceptable scientific standard.

# Response

**Published:** 2025-03-23

**Authors:** Frances Horgan

**Affiliations:** RCSI, Ireland

Dear Professor Hancock,

 Thank you for your very positive feedback and suggested changes to our protocol. We have addressed and made all of these changes as follows. 

 - It is important that people with lived experience are included in the summary of expert panel members in the abstract. I appreciate that their involvement is clearer later on in the manuscript (table 1), but a commitment to the full range of stakeholders should be explicit at the outset.

***Response - Our expert panel includes people with lived experience of stroke and carers. We have amended the abstract.***

 -The review question, in the second paragraph of page 3, is  general and does not include any reference to the population or context of life after stroke. Perhaps the addition of 'for people in their life after stroke' or similar would help here? 

***Response - thank you we are happy to make this change to ensure that the context is clearer.  We have added the suggested additional text so that the review questions are clear.***

 - A minor point, but suggest figure 1 legend simply says 'According to Pawson (ref) rather than "Pawson's work' 

***Response - we have changed the Figure 1 legend to According to Pawson (2002)***

 -the commitment to stakeholder engagement is laudable in the protocol, but I see no reference to carers. Carers are key stakeholders in life after stroke but seem to be absent from the expert panel- perhaps they will be included/represented by the SSOs but this could be clearer. 

**Response - *Our expert panel includes carers. We have added this detail in The expert panel section and in Table 1.***

 -a brief expansion of detail of the approach to coding and thematic analysis in the data analysis would enhance transparency and the interpretation of future work reporting the full study.

***Response – We will use the Braun and Clarke (29) thematic analysis framework to analyse the findings from each selected paper; (1) familiarity with the information, (2) generate codes, search for themes, (3) review the themes, (4) define and (5) names the themes and (6) produce the findings. Results and discussion sections will be coded in order to identify context, mechanism, outcome configurations in the findings. We have added this detail in the Data analysis section.***

 Frances Horgan on behalf of the author team
